# Screen time among Spanish university students with disabilities: a self-organizing maps analysis

**DOI:** 10.1186/s12889-019-7339-3

**Published:** 2019-07-24

**Authors:** Miquel Pans, Luis-Millán González, Joan Úbeda-Colomer, José Devís-Devís

**Affiliations:** Departament d’Educació Física i Esportiva, Universitat de València, C/ Gascó Oliag, 3, 46010 València, Spain

**Keywords:** Screen, Media, Disability, Adults, SOM

## Abstract

**Background:**

Screen time can play a significant role in the health and quality of life of people with disabilities. However, there is a lack of studies on this issue among people with disabilities, and even fewer in the university setting. Thus, the aim of our study was to explore the relationships between screen time, disability grade, body mass index (BMI), physical activity and sociodemographic variables (gender and socioeconomic status) in university students with different disabilities.

**Methods:**

A cross-sectional study was conducted on a sample of 1091 students with disabilities from 55 Spanish universities. Instruments used for data gathering were the Adolescent Sedentary Activity Questionnaire (ASAQ) and the International Physical Activity Questionnaire-Short Form (IPAQ-SF). A Self-Organizing Maps (SOM) analysis was carried out to explore the relationships between the variables under study.

**Results:**

Participants reported high values in overall screen time (5.45 h per day/week), with computers being the media most used (2.45 h per day/week). The SOM analysis showed slightly higher screen time values in women than men. People with a high disability grade spent less screen time than those with lower disability grade. Contradictory results exist when a group of men with the highest BMI had the highest screen time and the lowest physical activity (PA) while women with low BMI show the highest screen time and PA.

**Conclusions:**

Gender and disability grade played a moderating role in screen time among people with disabilities while BMI and PA do not play such a role.

## Background

Screen media technologies are accessible cultural goods available for consumption in our everyday life. Their use by people with disabilities is of growing interest due to their potential quality of life and health consequences [1–4]. Many studies have been published with therapeutic purposes, for either recovering body functions, expanding access to these technologies or improving social inclusion [4–8]. However, epidemiological studies addressing everyday screen time devoted to media usage (ST) by people with disabilities for leisure and work are scarce.

There are some exceptions with different results according to the disability analyzed. For instance, according to a study on adolescents with cerebral palsy, the participants spent an average of 4.18 h per day on ST. This study also found that men accumulate significantly more hours than women on ST [9]. Results obtained an ST average of 3 h per day in a sample of children with Autism Spectrum Disorder, TV viewing being the predominant source of screen time and showing non-gender differences [10]. Another study on children with intellectual disabilities, reported that they spent 1.36 h per day on ST [11]. This study also found a positive association between high ST values and high values of physical activity (PA). Conversely, no association was found between PA or socioeconomic status (SES) and ST in individuals with long-term illnesses [12]. Nor was body mass index (BMI) significantly associated with ST in youths with Autism Spectrum Disorder and chronic diseases [10, 13].

Some studies that compared adolescents with and without different disabilities did not find any differences in ST [13, 14], although different study did find significant differences in ST between the Autism Spectrum Disorder group and youths without disabilities, the former group presenting higher screen consumption time than their counterparts [10]. The differences found between hemophilic adolescents and their healthy counterparts indicated more time spent on screen media among the former [15]. A recent study also reported that adolescents with disabilities in 15 European countries spend more time on ST than their peers without disabilities [16].

ST can play a significant role in health and quality of life insofar as it contributes a great deal to the overall sedentary behavior associated with physical inactivity-related diseases (e.g. cardiovascular diseases, hypertension, Type 2 diabetes, obesity and metabolic syndrome) [17–19]. The study of ST in this population is thus of interest, since people with disabilities form an especially inactive group. University students with disabilities are an especially suitable target group, given their unique requirements for academic life. However, there is a lack of studies on ST in a nationwide sample of people with disabilities, and even fewer in the university setting. The purpose of this cross-sectional study is therefore twofold: firstly, to determine total ST of Spanish university students with disabilities and their partial usage of the technological media under study (television, video/DVD, computer, videogames and mobile). Secondly, to explore the relationships between ST (total and partial), the disability grade of the students and other variables (gender, SES, PA and BMI) by means of a Self-Organizing Maps (SOM) analysis.

## Method

### Participants

The *Guía de Atención a la Discapacidad en la Universidad* [20] was used to establish the population of students with disabilities since this is the most acknowledged institutional guide on disability care at Spanish universities. It includes relevant data such as the number of students with disabilities, disability care services contact information or the measures adopted at each university to favor inclusion (e.g. accessibility at the campus, curricular adaptations). In 2016 (the last academic year available during the data collection process) this guide included data from 76 universities and contained a total of 20,695 students with disabilities registered. An accessible population of 15,038 students was estimated during the study period. It was determined that 997 participants were needed for a statistically significant sample size (Confidence level = 95%; Population proportion = 50%; Margin of error = 3%), although finally 1,124 participants were enrolled. After excluding 33 respondents, who had reported implausible screen time (i.e. > 24 h per day) or had missing data on any ST question, 1,091 participants remained for the analyses. The participants showed a mean age of 40.15 (SD = 12.18), 529 were men, 557 were women and 5 did not specify their gender. They presented different types of disability: physical (e.g. spinal cord injury, cerebral palsy), mental disorder (e.g. Asperger syndrome, personality disorder), sensory (e.g. visual impairment, hearing impairment), chronic illness (e.g. fibromyalgia, osteoarthritis) and multiple disabilities (more than one type of disability concurrently). The severity of disability is expressed by the disability grade, another variable considered in this study (see Instrument and variables section), which refers to the percentage of activity limitation. This percentage is assessed by a multidisciplinary committee according to different criteria, established officially, on the impairment and participation restrictions, as well as complementary social factors (e.g. family environment, employment situation) applied to each type of disability. Table 1 shows the characteristics of the sample.

**Table 1:** Characteristics of Spanish university students with disabilities (*n* = 1091)

Variable	*n*	%
Gender		
*Male*	529	48.5
*Female*	557	51.1
*No answer*	5	0.5
Socioeconomic status		
*Low*	328	30.1
*Medium*	314	28.8
*High*	269	24.7
*No answer*	180	16.5
Disability grade		
*Low-Moderate (< 65% of disability)*	666	61.0
*High (≥65% of disability)*	420	38.5
*No answer*	5	0.5
Disability type		
*Physical disability*	468	42.9
*Mental disorder*	72	6.6
*Sensory disability*	135	12.4
*Chronic illness*	150	13.7
*Multiple disabilities*	232	21.3
*No answer*	34	3.1
Level of physical activity		
*Inactive-low*	429	39.3
*Medium*	434	39.8
*High*	228	20.9
BMI		
*Underweight*	43	3.9
*Normal*	509	46.7
*Overweight*	344	31.5
*Obesity*	177	16.2
*No answer*	18	1.6

### Procedure

The researchers established contact with the disability care services of the Spanish universities through a series of meetings and telephone contacts. The services which agreed to collaborate sent a questionnaire via e-mail to their students between April 2016 and February 2017. This indirect process was intended to protect the privacy and anonymity of the potential participants.

When the students accessed the survey there was a link to the written informed consent that explained the conditions of participation (e.g. voluntary and anonymous participation, confidentiality, right to refuse or abandon). Unless these conditions were accepted by clicking the proper box it was not possible to continue responding to the survey.

### Instruments and variables

The *Adolescent Sedentary Activity Questionnaire* (ASAQ) [21] was used in this cross-sectional study. This questionnaire has a good test-retest reliability [21] and was validated electronically [22]. The ASAQ has been widely used in different populations, including those with disabilities [15]. The subjects record the time spent on daily sedentary behavior during the preceding week in this self-reporting questionnaire. For the purposes of the study, only the sedentary screen variables from ASAQ were used, with added updated variables on mobile phone use and passive videogames.

PA was also measured by the *International Physical Activity Questionnaire-Short Form* (IPAQ-SF) [23], previously used in populations with disabilities [24].

Responses were recorded as continuous variables: a) TV viewing; b) video/DVD viewing; c) overall computer use (for play, communicating, or doing homework); d) mobile phone use; e) overall sedentary screen media (a + b + c + d); f) physical activity; and g) SES. The last variable was based on approximate monthly family income.

The categorical variables were gender, disability grade and BMI. The disability grade was the percentage of disability that appeared in their official medical report. In the Spanish welfare system, a 33% disability level is considered the minimum for access to all the social benefits. Two categories were thus established within the disability grade variable (low-moderate < 65% and high ≥ 65%), as is the usual practice [25]. Finally, they were asked for the estimated weight and height on which the BMI was calculated. Weight status was estimated using the BMI cut-off points recommended by the World Health Organization (WHO), as in previous studies on people with disabilities [26].

### Data analysis

Several analyses were performed after coding, cleaning and grouping the data. Descriptive statistics were obtained and expressed as means and standard deviations (SD). The main analysis was a technique based on artificial neural networks using unsupervised self-organizing maps (SOM), also known as Kohonen maps [27]. This technique has been widely used and various computer applications and functions have been developed in programming languages such as Matlab or R. SOMs have also been used successfully in different areas, including those with disabilities [28, 29].

The main objective of the SOM analysis was to transform an input signal pattern of arbitrary dimensions into a discrete two-dimensional map in a topologically ordered fashion. This type of analysis can be used to classify or detect relationships between a series of variables related to the problem. SOM analyses are also able to work with missing data [30].

Matlab R2012b (Mathworks Inc., Natick, USA) and the SOM toolbox (Version 2.0 beta) for Matlab were used for the SOMs. The process began with the construction of a network of neurons whose size depended on the number of cases in the analysis, according to the following equation: number of neurons ≈ 5 * √n; where n is the number of cases. The data matrix used as input had a total of 1091 cases or subjects. The network or grid had a rectangular shape with a size of 21 × 8 neurons high and wide, respectively. As the neurons were hexagonal in shape each of the central neurons had a total of 6 neighbors.

A value was then assigned for each of the input variables to each of the neurons or nodes (i.e. initialization). The SOM was initialized in two different ways, as follows. Random SOM initialization is used when the weight vectors started with a small random value. Linear initialization is used when the weight vectors are initialized in an orderly fashion along a linear subspace traversed by the two main eigenvectors of the input data series.

The weights initially assigned were modified throughout the training process. Two different training algorithms were applied (i.e. sequential and batch). In the training phase, each of the neurons competes to win each of the input vectors (*x*) or cases that make up the sample. The winning neuron in each case is the one with the smallest Euclidean distance between its vector of weights and the input vector. It should be noted that the vector inputs are normalized between 0 and 1 before beginning the training process. This was done in such a way that the scale of the variables did not influence the SOM training. Once the input data vector is assigned to a neuron, the weights of the winning neurons and neighboring neurons are modified and ordered topologically (i.e. ordering and convergence phases).

Eq.(1) shows the calculation used during the training of the neural network. The weights are modified after each iteration according to the differences between the initial weights and the input vector, the neighborhood function and the learning ratio.

*w*_*j*_(*n* + 1) = *w*_*j*_(*n*) + *η*(*n*)*h*_*j*, *i*(*x*)_(*n*)(*x* − *w*_*j*_(*n*)) Eq. (1).

Where *w*_*j*_ is the vector of weights of the *j*^*th*^ neuron, *η* is the learning ratio, *h*_*j*, *i*(*x*)_ is the neighborhood function and *x* is the input vector. The neighborhood function is used so that the winning neuron and its nearest neighbors adapt their weights to resemble the entry vector to a greater extent than the neurons furthest from the winner. Four different neighborhood functions were tested in the study: i) Gaussian, ii) Cut Gaussian, iii) Epanechicov and iv) Bubble.

The learning ratio is a high value for the first iterations and is progressively reduced to very small values. At the beginning of the training, the neuron weights undergo great changes and the changes become less pronounced as the process advances.

The entire process is repeated 100 times to increase the chances of finding the best solution to the problem. This is because the final result of the analysis depends on some random processes (e.g. initialization and input order of the input vector). Since we used two different training methods, four neighborhood functions and two initialization methods, 1600 SOM were finally obtained (i.e. 2 × 2 × 4 × 100).

The map selected showed the lowest product of the quantization error (0.25) multiplied by the topographic error (0.021). The quantization error expresses how well the neuron weight vector represents the cases that belong to the neurons. The topographical error is related to the position and value of the neurons. This error will be low when the nearby neurons resemble each other more than those that are further away.

Once the map with the least error had been established, the results were presented in different formats. Two areas of interest were established according to the disability grade. It is usual in this type of analysis to carry out a follow-up of some areas that appear on the maps. These areas of interest can be located mathematically or qualitatively. We selected two areas related to a greater degree of disability and are identified on the maps by a black triangle. To help in the statistical interpretation, the average values of the two areas of interest (High and Low-moderate disability grades) and of the effect of the comparison are shown by the Cohen d coefficient.

## Results

### Descriptive statistics

The participants reported an average ST of 5.45 h per day (SD = 3.71), mainly spent on computers, watching TV and using the mobile phone, while video/DVD and videogames were little used. The computer was the most used media by the respondents. Table 2 shows the ST descriptive data. The mean SES was €1948.19 (SD = 425.61), mean body mass index was 25.65 kg/m^2^ (SD = 5.79), and mean PA was 1938.30 MET-minutes/week (SD = 2717.95).

**Table 2:** Descriptive statistics for daily use of screen media by university students (*n* = 1091)

	Mean	(SD)
TV	1.28	(1.22)
Video/DVD	0.42	(0.73)
Computer	2.65	(2.33)
Video Games	0.08	(0.35)
Mobile	1.04	(1.40)
Screen media usage	5.45	(3.71)

*Data are expressed in mean of hours/day*

### Self-Organizing Maps

**Fig. 1:**
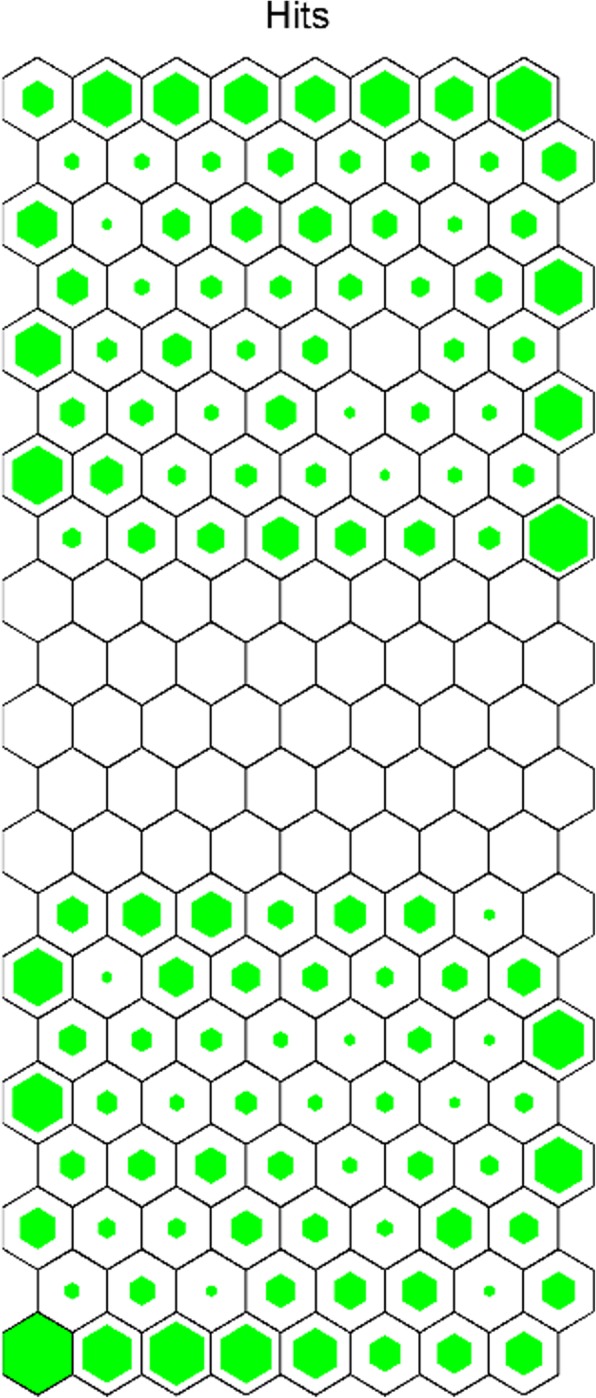
Hit map. The green area represents the number of subjects within each neuron. The larger the size, the greater the number of subjects

Because of the SOM analysis, 11 maps of the study variables emerged, as well as a hit map. The latter helped to explain how the subjects of the sample were distributed. The distribution of participants was in the upper and lower parts of the map, especially around the borders (see Fig. 1). A group of empty neurons appeared in the center because the mathematical process tried to avoid topographical errors and occasionally left empty spaces.

**Fig. 2:**
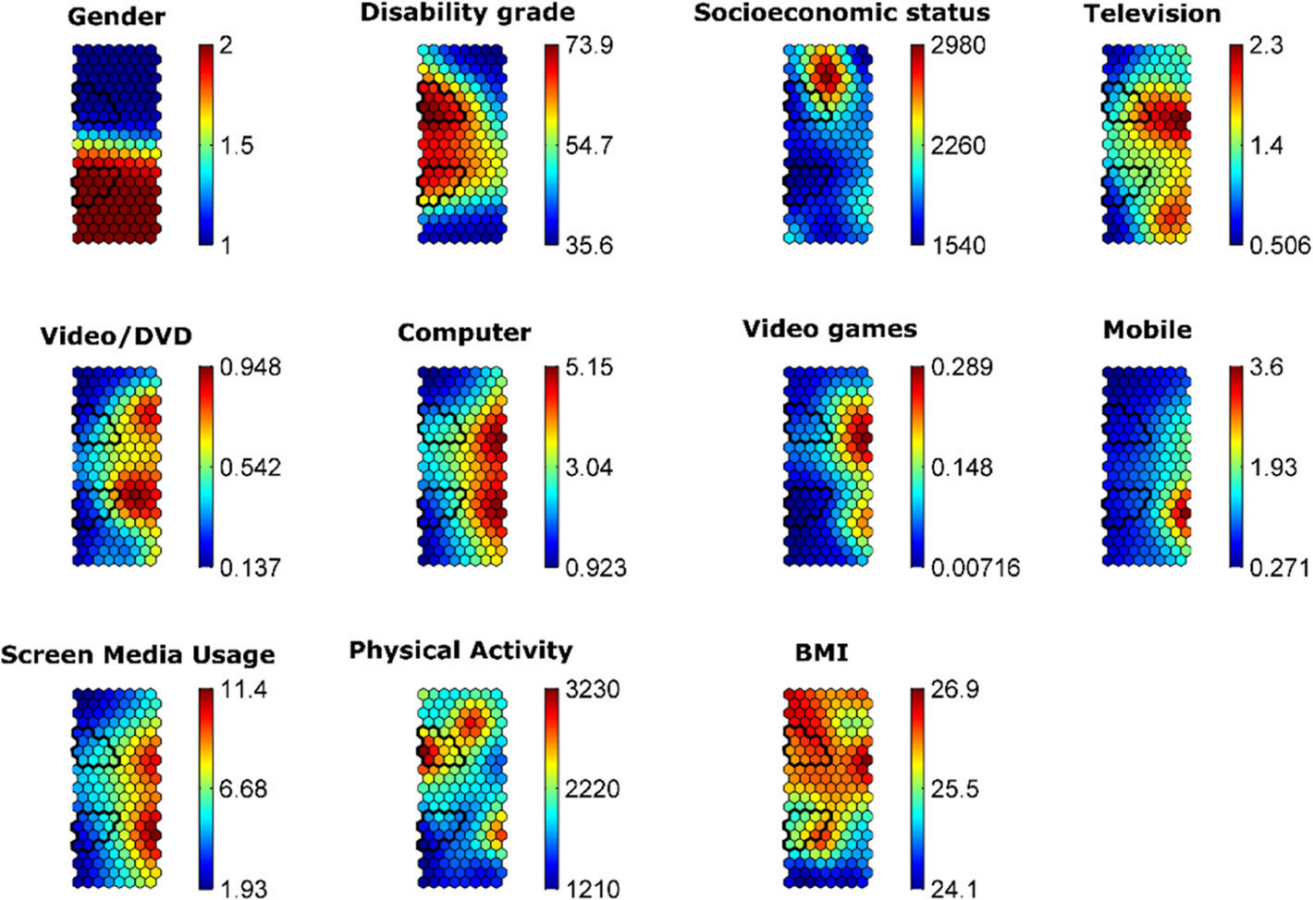
Component planes of the registered variables. From left to right and top to bottom are the component planes of the variables: Gender (male = 1, female = 2); Disability grade (%); Socioeconomic Status (€); Television use (hours*day); Video/DVD use (hours*day), Computer use (hours*day), Mobile phone use (hours*day), Video games use (hours*day) and the total Screen Media Usage (hours*day); Physical Activity (mets) and BMI = Body Mass Index (kg·m^2^). The black triangles around the interest zones indicate the disability grade

The component planes or maps showed the values obtained for each variable and indicated the intensity or number of participants within each node (see Fig. 2). High values were shown in red and the low values in blue. The scale at the right of each map explains the component planes. According to the SOM analysis, each participant was located in the same place in each map. The analysis included a comparison of the topological relationships among the planes.

The media and gender maps showed that the time devoted to overall media usage was slightly higher in women than in men. Although computers were the media most used by both genders, there were some gender differences in other screen media. For instance, men spent more time than women watching TV and playing videogames, while women spent more time on video/DVD and mobile phones than men did.

The disability grades were distributed with the highest levels (in two black triangles) in the center-left of the map and the highest concentration in the upper triangle (men) than in the lower triangle (women). This distribution revealed that men and women with the highest disability grades were the ones who spent least ST.

The SOM analysis also showed topological relationships between other maps; some men with high BMI presented the lowest levels of ST and highest PA, while the small group with the highest BMI values presented the highest ST values and the lowest PA. On the other hand, the women with the lowest BMI levels showed the least ST and the lowest PA. Only the small group of women with the highest BMI presented low ST and PA values, while the small low BMI group showed the highest ST values and PA.

The descriptive statistics of each variable by disability grade are shown in Table 3. The mean and SD of the high disability (i.e. the interest zones surrounded) and low-moderate disability (i.e. unenclosed area) groups were calculated for each variable. In general, ST is lower in people with high disability grades (effect size of d = 0.53) than those with low-moderate disabilities. The high disability grade group spent slightly less time than the low-moderate group on mobile phone and computers.

**Table 3:** Descriptive statistics of each variable according to disability grade

	Low-moderate disability grade (Unenclosed area)	High disability grade (Interest zones)	Cohen’s d
Socioeconomic status	1697.07 (1583.54)	1372.07 (1049.61)	0.24
TV	1.25 (1.25)	1.13 (1.13)	0.11
Video/DVD	0.43 (0.76)	0.26 (0.51)	0.27
Computer	2.67 (2.50)	1.80 (1.51)	0.42
Video Games	0.09 (0.38)	0.03 (0.14)	0.21
Mobile	1.09 (1.50)	0.54 (0.65)	0.48
Screen media usage	5.46 (4.04)	3.66 (2.53)	0.54
PA (total MET)	1593.94 (2398.26)	2060.70 (3286.29)	0.16
BMI	25.27 (6.49)	25.19 (6.85)	0.01

*Effect size can be interpreted according to Cohen’s ‘d’ statistic: d = 0.20: small; d = 0.50: moderate; d = 0.80: large*

## Discussion

The results obtained indicate that Spanish university students with disabilities have a high overall use of screen media, close to an average 6 h per day, compared to between 2.5 and 4 h ST per day in the general young population [31] or the 2.4 h reported by college students [32] with no declared disabilities. However, a study on the use of media among 8 to 18-year-old USA students reported 7.5 h per day [33]. This is probably due to the general evolution of the technological culture in western societies and to the particular characteristics of the population under study. The recent rapid growth in the use of technology and media, especially in mobile phones, could explain the differences between our study and previous research on the general young population. Since our sample was made up of university students, it is also possible that the high level of media use was due to academic requirements, as was found in studies on female university students with no declared disabilities [34], which is consistent with the finding that the computer is the most frequently used media in this study.

However, the high ST values among our participants could also be to compensate for the possible reduced face-to-face social relationships in their daily lives. Although, this hypothesis will need to be verified in future studies, there is some evidence in our data to support it. For instance, the total ST is closer to the 4 h per day obtained by a research in a sample of cerebral palsy adolescents [9] than to other studies on participants without disabilities [31, 32]. Several studies that explicitly compared ST in people with and without disabilities found higher values in the disabled than in the non-disabled sample [10, 11, 15, 16].

Nevertheless, this hypothesis needs to be nuanced by participants’ disability grade. According to our results, this factor may play a moderating role in the time our participants devoted to screen media, since the higher the disability grade, the less time they devoted to particular and total screen media. This is probably due to the lack of expensive assistive technologies accessible to people with disabilities [35]. Even though recent improvements and adaptations have been made in these devices, they may still be insufficient [36, 37]. In fact, the small sized and accessible mobile phone is not the best option for people with severe disabilities [38].

The present results also show differences in overall and particular screen media types. For instance, women spent slightly more time on overall screen media than men, while other studies on children and adolescent samples found no differences by gender or with men, who are the ones who spend most time on this device [9, 10]. The different ages of the samples in the previous studies may have influenced these differences in overall screen media. However, a trend of equality by gender has been observed in the non-disable young population as new generations are introduced to electronic culture [33]. In this sense, the gender differences observed in our study by type of screen media are similar to those of the general population in developed countries, since men spend more time on TV and videogames than women, and the latter spend more time on mobile phones [33, 39]. These results are in line with previous studies on the general population, which suggest men are keener on technology-based activities such as videogames and women on social activities linked to screen media [31, 40].

Other topological relationships from the SOM analysis gave contradictory results, since both men and women with different BMI showed different patterns of ST and PA. The small group of men with the highest BMI values had the highest ST values and the lowest PA, which seems to be in line with the risk of becoming obese, as has been suggested in the literature [40]. On the other hand, another group with high BMI values reveals the lowest ST values and the highest PA. A contra-intuitive relationship was also found in women participants with low BMI and the highest ST and PA. These results suggest that the explanation for the risk of obesity in high ST values in the normal population is less clear than in people with disabilities, probably due to factors other than ST affecting high levels of BMI. Therefore, ST may play a more positive role in the lives of people with disabilities [41] than in the non-disabled.

Finally, according to our results, the disability grade interacts in a complex way with ST and PA. Although men with the highest disability grade get high values on PA and less on ST, there is also a group of men with a low disability grade with high PA and low ST, as well as another low disability group with low PA and little ST. Only a small group of women in the low disability grades showed high values of both PA and ST. Further research is needed to determine how PA may be affected by other factors other than ST and disability grade.

Our paper has certain limitations that should be pointed out. Firstly, an on-line questionnaire may collect less reliable data than a face-to-face questionnaire. It is more frequent to find missing answers or misunderstandings in completing questions in the former than the latter. Secondly, self-reported measures, as used in this study, have been criticized in previous studies because they biased values for participants’ classification purposes. For instance, height has been overestimated and weight has been underestimated in obtaining BMI values, specially observed among overweight healthy adults and people with different pathologies [42–44]. However, discrepancies with direct measures of BMI were small and self-reported measures, if accurate, still provide a simple and economical method for body weight purposes [44, 45]. ASAQ may also be limited by both recall and a social desirability bias. However, the validity and reliability obtained in the methodological studies, as mentioned above, as well as the tertiary level of our participants, do not suggest recall problems when answering the questionnaire. We also highlight that our descriptive data show high values in SD and it is probably due to the heterogeneity of our sample. Therefore, we decided to avoid classical statistical analyses (e.g. statistical inference) in favour of an artificial neural network analysis, which allows working with atypical distributions.

The overall results of the present study may contribute to future interventions on ST among people with disabilities. The general literature indicates that excessive daily ST use can be harmful to people’s health [46, 47], but this finding needs to be balanced with the potential social benefits for the participants’ social limitations. According to our data, high ST values are not always related to high levels of BMI or the risk of obesity and may play a positive role in enhancing social and communicative skills and processes with other persons, which also have health benefits for people with disabilities. These potential benefits would thus be increased if more assistive technologies were accessible to them, instead of emphasizing their control or minimizing their use.

## Conclusions

This paper is the first to study ST on university students with different disabilities in a nationwide Spanish sample. It is also the first to address this issue using a SOM Analysis to explore topological relationships between ST and disability grade as well as other variables of interest such as gender, SES, PA and BMI. The results indicate that Spanish university students with disabilities show high values of ST, being slightly higher in women than in men. Although computers are the media most used by both genders, men spend more time than women watching TV and playing videogames, while women spend more time on video/DVD and mobile phones than men. The disability grade plays a moderating role, determining the ST since people with superior disability grade spend less ST than those with inferior grades. Contradictory results exist in the relationships among ST, BMI and PA. A small group of men with the highest BMI values had the highest ST values and the lowest PA while another group with high BMI values reveals the lowest ST values and the highest PA. A contra-intuitive relationship is also found in women participants with low BMI and the highest ST and PA. Even so, the findings from this study contribute to fill the lack of knowledge in the scientific literature about ST in university students with disabilities. In addition, this knowledge is useful for future intervention programs among people with disabilities, balancing the negative consequences of excessive ST with its potential social benefits.

## Data Availability

The datasets generated and/or analysed during the current study are not publicly available because in the informed consent form the authors did not indicate that they would share data to third parties, but are available from the Ethics Committee of Universitat de València on reasonable request.

## Abbreviations

ASAQ: Adolescent Sedentary Activity Questionnaire
BMI: Body mass index
IPAQ: International Physical Activity Questionnaire
PA: Physical activity
SD: Standard deviations
SES: Socioeconomic status
SOM: Self-Organizing Maps
ST: Screen time
